# Supplementary material for: Novel Multifunctional Luminescent Electrospun Fluorescent Nanofiber Chemosensor-Filters and Their Versatile Sensing of pH, Temperature, and Metal Ions
Source: Polymers (Basel). 2018 Nov 13;10(11):1259. doi: 10.3390/polym10111259 (PMC6401977; doi:10.3390/polym10111259)
Supplement: Supplementary file 1 [file polymers-10-01259-s001.pdf]

# Supplementary Materials: Novel Multifunctional Luminescent Electrospun Fluorescent Nanofiber Chemosensor-Filters and Their Versatile Sensing of pH, Temperature, and Metal Ions

**Bo-Yu Chen, Yen-Chen Lung, Chi-Ching Kuo, Fang-Cheng Liang, Tien-Liang Tsai, Dai-Hua Jiang, Toshifumi Satoh and Ru-Jong Jeng**

### Synthesis of the Fluorescent Probe (RhBN2) and Fluorescent Monomer (RhBN2AM)

The synthetic route was shown in Scheme 2a. The synthesis of a fluorescent probe, RhBN2, was performed according to a previous report [5]. Ethylenediamine (4.8 g, 10 mmol) was dissolved in 30 mL of ethanol. The mixture was immersed in an oil bath and refluxed for 20 h until the fluorescence of the solution disappeared. The solvent was removed under reduced pressure. $CH_2Cl_2$ (300 mL) was then added to the product, and the solution was washed with water several times and dried over an anhydrous magnesium sulfate. The filtrate was collected and the solvent was removed under reduced pressure, to obtain a pale-pink powder.

In a two-necked round-bottomed flask, RhBN2 (2 g, 4.14 mmol) was dissolved in 80 mL of THF, then TEA (2.88 mL) and excess acryloyl chloride were dropped into the stirred solution at 0 °C, and the reaction mixture was stirred at room temperature for 1 day. The solution was concentrated, then the resulting solid was purified by column chromatography using dichloromethane/methanol (20:1, *v*/*v*) as eluent. The chemical structure of RhBN2AM was characterized using $^1$H NMR (Figure S1a) and ESI-MS (Figure S1b). $^1$H NMR (300 MHz, $CDCl_3$, δ, ppm; Figure S1a): 6.24–7.88 (10H, aromatic hydrogen), 7.12 (1H, f), 6.15 (1H, g), 6.04 (1H, e), 5.55 (1H, e), 3.31 (10H, b, c), 3.13 (2H, d), 1.15 (12H, a). ESI-mass (Figure S1b): m/z calcd for $C_{33}H_{39}N_4O_3+H^+$([M+H$^+$]), found, 539.3010.

### The Photoluminescence (PL) Spectra Based on ES Nanofiber for pH and $Hg^{2+}$ Sensing

Photoluminescence (PL) spectra were measured to study photophysical properties. PL experimental data were recorded using a Fluorolog-3 spectrofluorometer (Horiba Jobin Yvon). To ensure that the beam would excite the same point on the prepared samples at each measurement, the ES nanofibers were fixed in cuvettes by using an adhesive tape, and the cuvette was filled with an aqueous metal ion solution at $10^{-7}$ to $10^{-3}$ M or acidic and basic aqueous solutions. Each measurement was maintained for 10 min to ensure that an equilibrium of the chelating reaction was reached. All PL spectra of the ES nanofibers were recorded using the Fluorolog-3 spectrofluorometer at an excitation wavelength of 540 nm, as described in our previous studies [28–37].

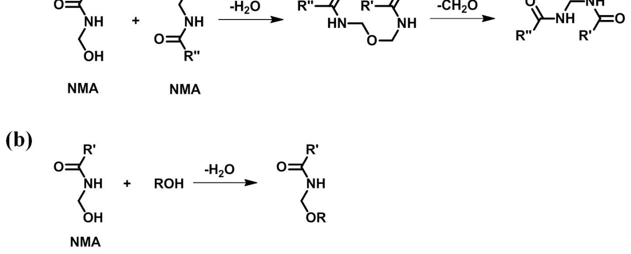


**Scheme S1.** Potential (**a**) NMA-NMA and (**b**) NMA-hydroxyl cross-links.

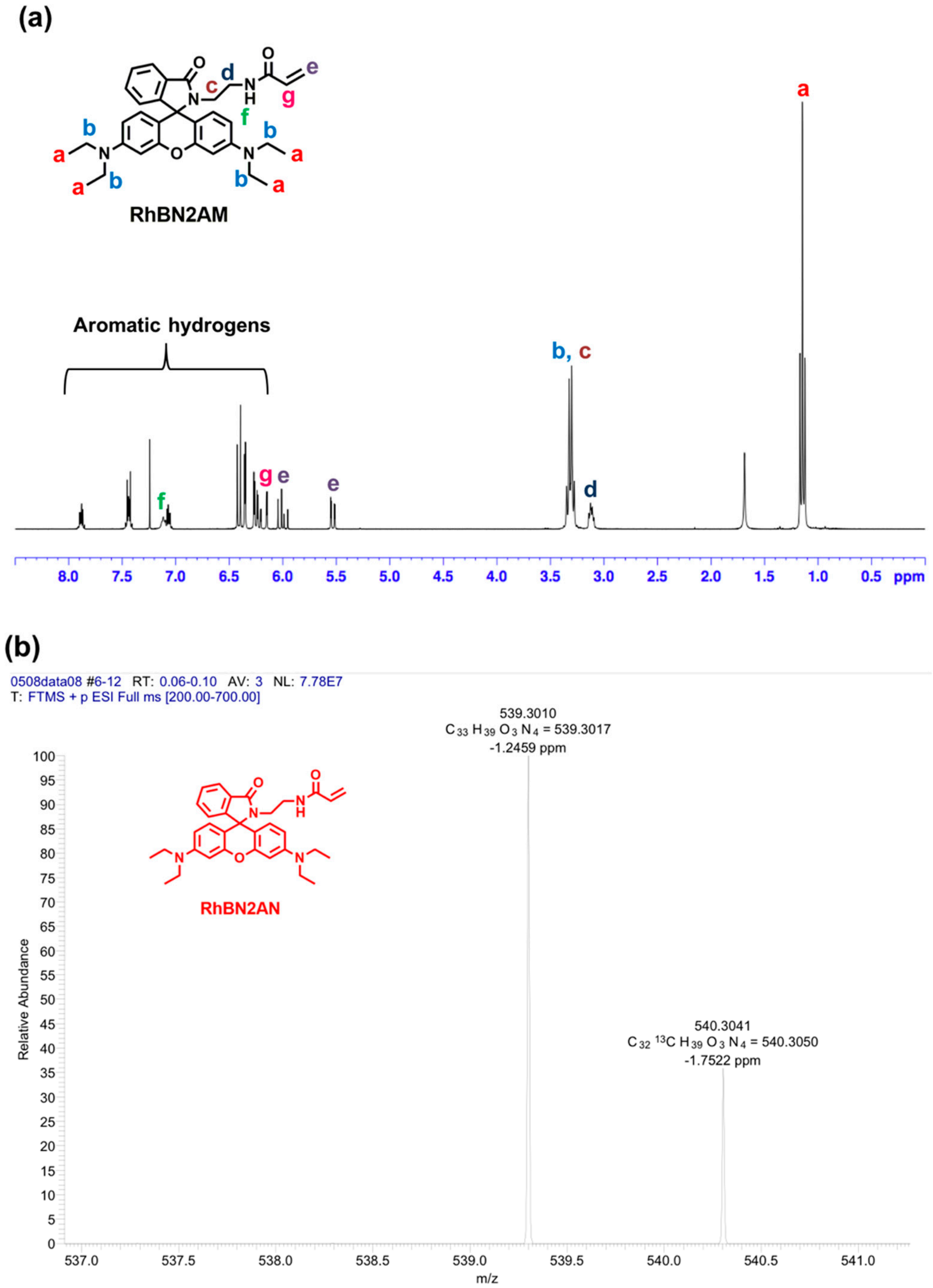


**Figure S1.** (**a**) $^{1}$H NMR spectrum of RhBN2AM in $CDCl_3$. (**b**) ESI-MS spectrum of RhBN2AM.

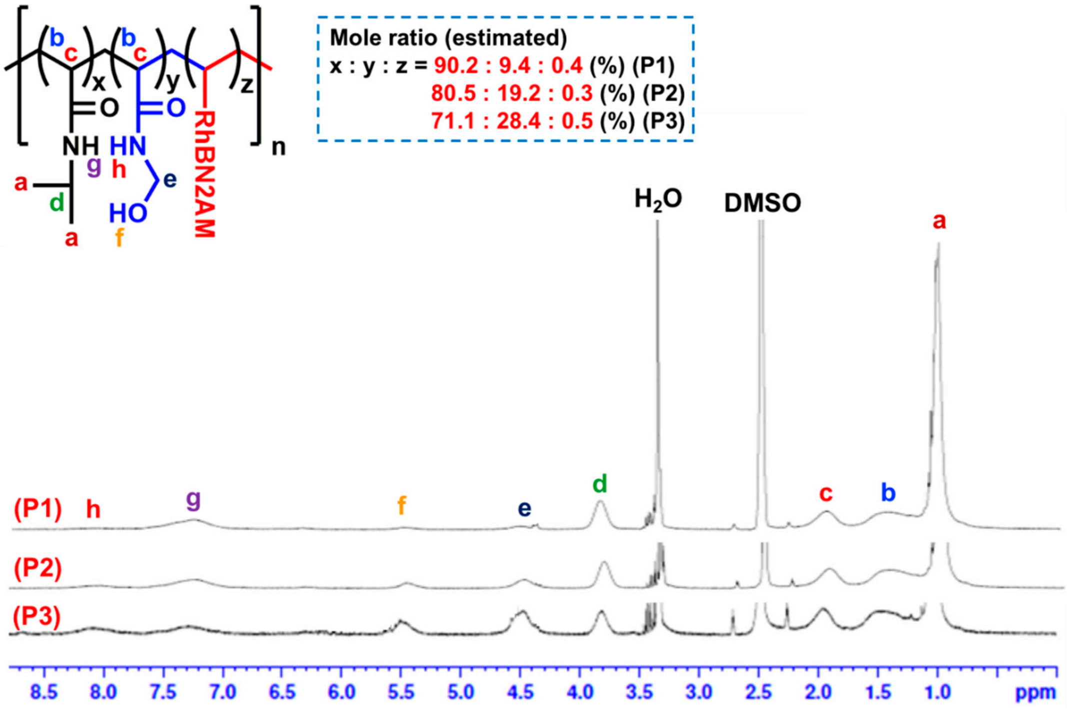


**Figure S2.** $^{1}$H NMR spectra of **P1**, **P2,** and **P3.**

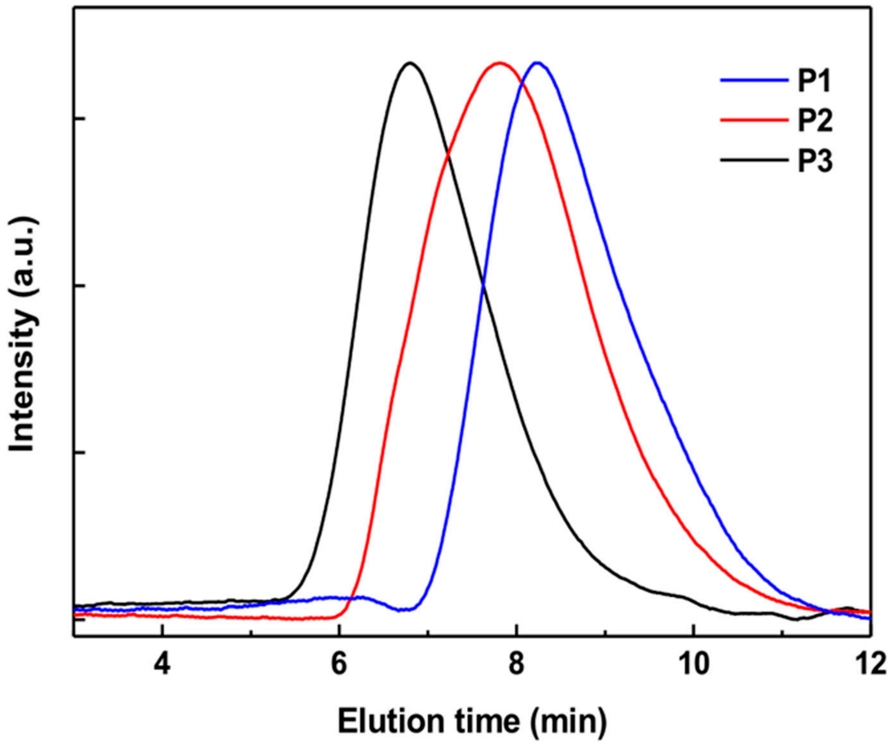


**Figure S3.** GPC profiles of **P1**, **P2**, and **P3**.

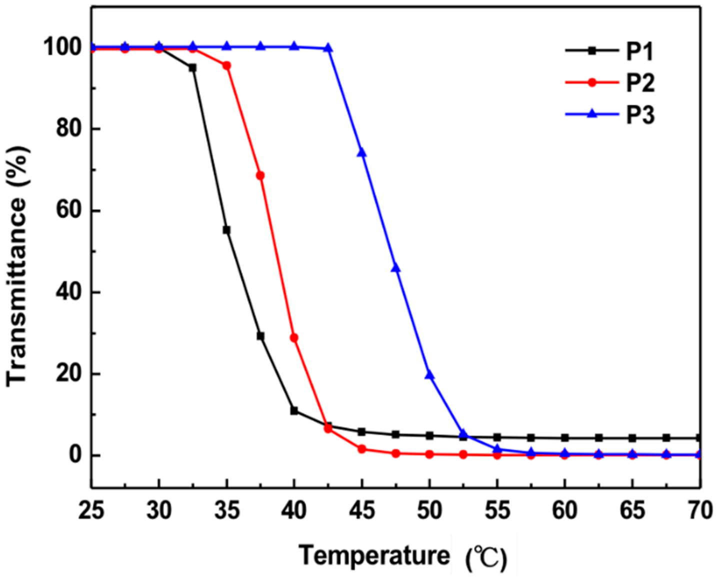


**Figure S4.** Variations in optical transmittance of **P1**, **P2**, and **P3** in water at temperatures from 25 to 70 °C.

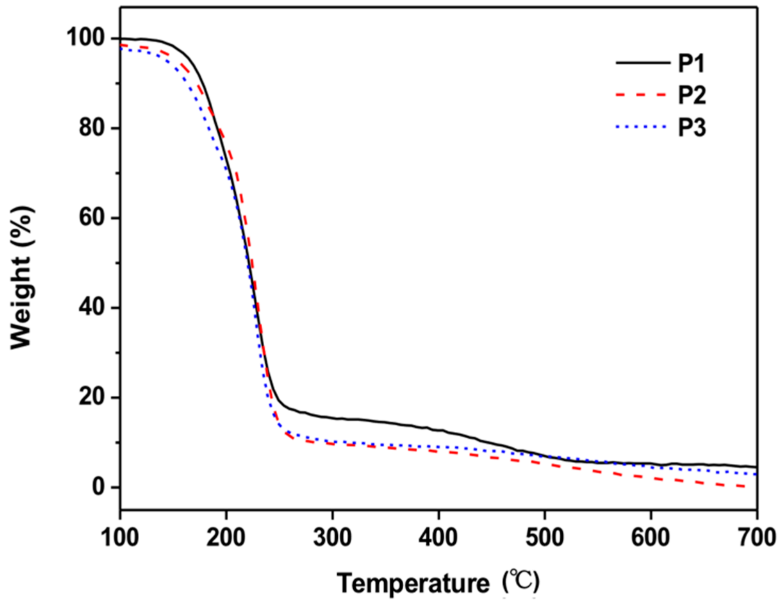


**Figure S5.** TGA curves of **P1**, **P2**, and **P3**.

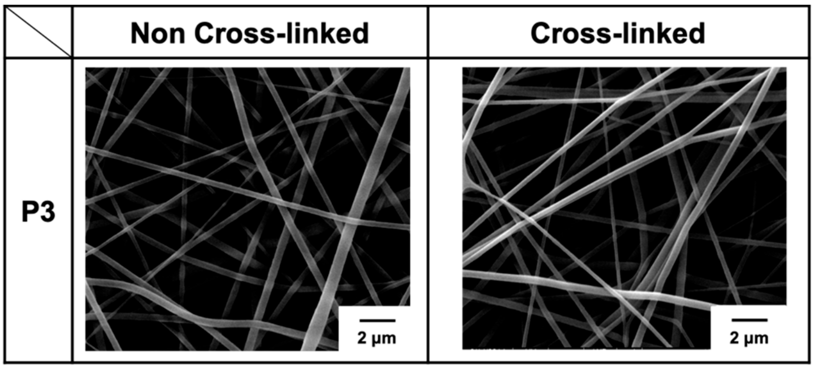


**Figure S6.** Comparison of the average diameter of non-cross-linked and cross-linked **P3** ES nanofibers.
